# *Celastrus orbiculatus* Thunb. extracts and celastrol alleviate NAFLD by preserving mitochondrial function through activating the FGF21/AMPK/PGC-1α pathway

**DOI:** 10.3389/fphar.2024.1444117

**Published:** 2024-08-05

**Authors:** Junli Xue, Yunchao Liu, Boyan Liu, Xiubin Jia, Xinsheng Fang, Shucun Qin, Ying Zhang

**Affiliations:** ^1^ Taishan Institute for Hydrogen Biomedicine, The Second Affiliated Hospital of Shandong First Medical University and Shandong Academy of Medical Sciences, Tai’an, Shandong, China; ^2^ School of Pharmaceutical Sciences, Shandong First Medical University and Shandong Academy of Medical Sciences, Ji’nan, Shandong, China; ^3^ College of Agronomy, Shandong Agricultural University, Tai’an, Shandong, China

**Keywords:** *Celastrus orbiculatus* Thunb., celastrol, NAFLD, mitochondrial function, FGF21/AMPK/PGC-1α

## Abstract

**Objective:**

Non-alcoholic fatty liver disease (NAFLD) is a prevalent chronic liver disease globally, characterized by the accumulation of lipids, oxidative stress, and mitochondrial dysfunction in the liver. *Celastrus orbiculatus* Thunb. (COT) and its active compound celastrol (CEL) have demonstrated antioxidant and anti-inflammatory properties. Our prior research has shown the beneficial effects of COT in mitigating NAFLD induced by a high-fat diet (HFD) in guinea pigs by reducing hepatic lipid levels and inhibiting oxidative stress. This study further assessed the effects of COT on NAFLD and explored its underlying mitochondria-related mechanisms.

**Methods:**

COT extract or CEL was administered as an intervention in C57BL/6J mice fed a HFD or in HepG2 cells treated with sodium oleate. Oral glucose tolerance test, biochemical parameters including liver enzymes, blood lipid, and pro-inflammatory factors, and steatosis were evaluated. Meanwhile, mitochondrial ultrastructure and indicators related to oxidative stress were tested. Furthermore, regulators of mitochondrial function were measured using RT-qPCR and Western blot.

**Results:**

The findings demonstrated significant reductions in hepatic steatosis, oxidative stress, and inflammation associated with NAFLD in both experimental models following treatment with COT extract or CEL. Additionally, improvements were observed in mitochondrial structure, ATP content, and ATPase activity. This improvement can be attributed to the significant upregulation of mRNA and protein expression levels of key regulators including FGF21, AMPK, PGC-1α, PPARγ, and SIRT3.

**Conclusion:**

These findings suggest that COT may enhance mitochondrial function by activating the FGF21/AMPK/PGC-1α signaling pathway to mitigate NAFLD, which indicated that COT has the potential to target mitochondria and serve as a novel therapeutic option for NAFLD.

## 1 Introduction

Non-alcoholic fatty liver disease (NAFLD) is a chronic liver condition marked by the steatosis and accumulation of lipid droplets in the liver, which is not linked to alcohol consumption. Recently, this condition has been reclassified as metabolic dysfunction-associated fatty liver disease (MAFLD) (Eslam et al., 2020) and even metabolic dysfunction-associated steatotic liver disease (MASLD) (Rinella et al., 2023) to emphasize the key role of metabolic disorders. NAFLD encompasses a broad range of manifestations, ranging from steatosis, steatohepatitis, fibrosis to cirrhosis, and is a leading cause of hepatocellular carcinoma and the primary indication for liver transplantation in western nations (Fang et al., 2023). With an estimated global prevalence ranging from 25% to 45%, NAFLD is the most prevalent chronic liver disease (Cheng et al., 2022). NAFLD frequently leads to the development of cardiovascular diseases, malignant tumors, and end-stage liver disease, ultimately becoming the primary cause of mortality in affected individuals. The impact of NAFLD extends beyond the individual patient, affecting their quality of life and placing a significant burden on their families and society. Despite the rising prevalence of NAFLD, therapeutic options are constrained, primarily focusing on dietary modifications and physical activity, which are often not sustainable. Currently, there are no approved medications for the treatment or prevention of NAFLD (Dong et al., 2023), highlighting the necessity for further research and development in this area.

While the exact pathophysiology of NAFLD remains incompletely elucidated, the “double hit” theory has traditionally been regarded as a classical pathogenic mechanism. This theory posits that the initial hit involves the accumulation of excess lipids and insulin resistance in hepatocytes, leading to subsequent inflammation and fibrogenesis (Day and James, 1998). As research advances, it is increasingly apparent that the pathogenesis of NAFLD is more intricate than initially believed, and the “multiple hit” hypothesis is now gaining prominence. This hypothesis encompasses a range of pathological mechanisms, including insulin resistance, oxidative stress, lipotoxicity, inflammation, endoplasmic reticulum stress, apoptosis, and gut-microbiota dysfunction (Guo et al., 2023). It should be noted that mitochondrial damage is a crucial factor in various physiological processes, such as morphological changes, abnormal fatty acid and energy metabolism, lipid oxidative stress, and inflammation (Mansouri et al., 2018). Research has indicated that individuals with steatosis exhibit mitochondrial dysfunction and oxidative stress in liver tissues, characterized by diverse manifestations of mitochondria ultrastructure damage, ATP depletion, heightened reactive oxygen species (ROS) production, and β-oxidative damage (Wells et al., 2008; Ramanathan et al., 2022). Moreover, compromised mitochondrial biogenesis leading to failure of mitochondrial quality control is a significant contributing factor. Consequently, the implementation of mitochondria-targeted therapy to enhance mitochondrial function has emerged as a vital approach in the management of NAFLD.

AMP-activated protein kinase (AMPK), a prominent metabolic energy sensor, can detect cellular energy levels by directly interacting with adenine nucleotides. It is activated in response to a decrease in ATP levels and an increase in the adenosine monophosphate (AMP)/ATP ratio. Upon activation, AMPK alters metabolic processes by enhancing catabolism and reducing anabolism through the phosphorylation of key proteins in various pathways, including lipid and mitochondrial homeostasis (Herzig and Shaw, 2018). As a key downstream effector of AMPK, peroxisome proliferator-activated receptor γ co-activator-1α (PGC-1α) serves as a central regulator. It can be interacted and phosphorylated by AMPK, resulting in the enhancement of transcriptional activity of PGC-1α (Abu Shelbayeh et al., 2023). Studies have indicated that oxidative stress and AMPK/PGC1α-mediated mitochondrial dysfunction are involved in the pathological alterations of in lipid metabolism associated with NAFLD (Dahlhoff et al., 2014; Yang et al., 2021). AMPK activation can modulate glucose and fatty acid metabolism, mitochondrial function, production of ROS and pro-inflammatory cytokines, ultimately impeding the progression of NAFLD (Lin et al., 2021; Fang et al., 2022). Meanwhile, in mice with liver-specific AMPK knockout, exacerbated liver lipid accumulation, steatosis, fibrosis, and inflammation were observed (Zhao et al., 2020). Additionally, the relative expressions of mRNA and protein of PGC-1α were found to decrease in mice/rats with NAFLD (Wen et al., 2021; Meng et al., 2024; Wang et al., 2024). Consequently, targeting this pathway may offer a novel and promising approach for the treatment of NAFLD (Zhang et al., 2023).

In recent years, traditional Chinese medicine has gained increasing prominence in the treatment of NAFLD. Various traditional Chinese medicines, such as *Alisma plantago-aquatica* Linn. (Ho et al., 2019), *Gastrodia elata* Bl. (Wan et al., 2021), and some monomers like resveratrol (Teng et al., 2019), ginsenoside Rg1 (Liu et al., 2018), oxymatrine (Xu et al., 2020) and puerarin (Kang et al., 2015), have been found to activate AMPK and improve NAFLD. *Celastrus orbiculatus* Thunb. (COT) is a shrub plant belonging to the genus *Celastrus* in the Celastraceae family. It is a more sustainable and eco-friendlier source of medicine compared to synthetic drugs. This conventional herbal remedy exhibits numerous beneficial properties, including antioxidant and anti-inflammatory effects, and holds promise for a wide range of therapeutic uses in the management of conditions such as cancer (Feng et al., 2023), cardiovascular diseases (Li et al., 2016; Li et al., 2022), and chronic obstructive pulmonary disease (Yang et al., 2020). Of particular interest, several studies have shown that the important bioactive ingredient celastrol (CEL) may modulate lipid metabolism by inhibiting lipogenesis or promoting lipolysis, suggesting potential benefits against the progression of NAFLD (Zhang et al., 2017; Zhang et al., 2019; Fan et al., 2022b). Our team has previously demonstrated that administration of COT extract effectively mitigated high-fat diet (HFD)-induced NAFLD in guinea pigs by reducing hepatic lipid levels and suppressing oxidative stress (Zhang et al., 2013). However, further investigation is needed to elucidate the specific mechanisms, particularly those pertaining to mitochondria, responsible for the protective effects of COT on NAFLD.

This study investigated the anti-NAFLD effects of COT extract and CEL through both *in vivo* and *in vitro* experiments. Key regulators associated with mitochondrial biogenesis and function, with PGC-1α as the core, were detected following treatment with COT extract or CEL. The findings of this study, along with the elucidated mitochondrial regulatory pathways, could offer promising dietary supplements and drugs for the treatment of NAFLD.

## 2 Materials and methods

### 2.1 Preparation of *Celastrus orbiculatus* Thunb. extract

The dried slices of the root of COT were collected from the Taishan Region in Shandong Province, China. They were pulverized into powders and screened through the 50-mesh sieve. The harvest dried coarse powder (100 g) was subjected to three extractions with 95% ethanol under reflux conditions for a duration of 3 h each. Subsequently, the filtrate underwent evaporation in order to procure the extract derived from the roots of COT. The quantification of CEL content was conducted using high-performance liquid chromatography (HPLC). A standard curve was constructed according to the concentration of the standard solution and the corresponding measured absorbance values. Subsequently, the concentration of the unknown sample was determined based on its absorbance reading.

### 2.2 Animals and experimental design

Six-week-old specific pathogen free level male C57BL/6J mice weighing 16–18 g were procured from Pengyue Experimental Animal Breeding Co., Ltd. [Jinan, China; license No. SCXK (Lu) 20190003]. The mice were housed in cages with unrestricted access to food and water in a standard environment maintained at a constant temperature of 20°C–24°C and relative humidity of 50%–60%, with a 12-h dark/light cycle. All animal experiments were approved by the Ethics Committee of the Second Affiliated Hospital of Shandong First Medical University (Ethical No. 2022A027) and performed strictly according to the Guide for the Care and Use of Laboratory Animals.

After a 1-week acclimation period with a standard diet, mice were randomly divided into eight groups (n = 10 per group) and received various treatments. The control group (CON) continued the standard diet, while the remaining groups were fed HFD in order to induce NAFLD. The HFD consisted of 24.2%, 40.1%, and 27.4% protein, carbohydrate, and fat, respectively. In contrast, mice in the drug groups were administered the following treatments: the statin group (STA) received 20 mg/kg/day of simvastatin (Merck Sharp and Dohme Pty. Ltd.) via gavage (dissolved in 0.5% CMC-Na), the CL, CM, and CH groups were administered 1, 3, and 5 mg/kg/day of CEL, respectively. According to our previous work (Zhang et al., 2013), the EL and EH groups were given 2.5 and 10 g/kg/day of COT extract, respectively. Over the course of the 12-week intervention, weekly measurements of body weight (BW) were taken. Following this period, all mice were euthanized, and samples of serum, liver, and visceral fat were collected for further experiments.

### 2.3 Oral glucose tolerance test (OGTT)

In the 11th week of the study, OGTT was conducted on groups of mice consisting of six individuals each, following a 12-h fasting period. The mice were orally administered a 50% glucose solution at a dosage of 2 g/kg body weight, after which blood samples were obtained through tail vein incision at 0, 30, 60, 90, and 120 min to measure blood glucose levels. Blood glucose curves were plotted based on the recorded values at each time point, and the areas under the curve (AUC) were calculated.

### 2.4 Cell culture and treatments

The HepG2 cell line obtained from Pricella Biotechnology Co., Ltd. (Wuhan, China) was cultured in DMEM high-glucose basal medium supplemented with 10% fetal bovine serum (FBS) under standard conditions of 95% air and 5% CO2 at 37°C. The NAFLD cell model was established by supplementing sodium oleate (NaOL) to the culture. HepG2 cells in the logarithmic growth phase were harvested and seeded at a density of 1 × 10^4^ cells/well in a 6-well plate after digestion. After 24 h of incubation, the cells were divided into nine groups for experiments. The control group (CON) consisted of HepG2 cells that did not undergo any treatment. The remaining groups were subjected to induction of high fat cells using NaOL (0.5 mM), and the simvastatin group (STA, 10 μM) served as a positive control. As our previous study (Zhang et al., 2016), the drug groups (CL, CM, CH) were co-treated with varying concentrations (0.1, 0.2, 0.4 μg/mL) of CEL, while the extract groups (EL, EM, EH) were co-treated with different concentrations (50, 100, 150 μg/mL) of COT extract. After 24 h, all cells were collected for further experiments.

### 2.5 Biochemical analyses

TG, TC, and/or LDL-C levels in serum and cell supernatant were quantified using commercially available kits (Jiancheng Bioengineering Institute, Nanjing, China) following the manufacturer’s protocols. Levels of ALT and AST in liver homogenates or serum were determined using specific detection kits (Jiancheng Bioengineering Institute, Nanjing, China). Enzyme-linked immunosorbent assay (ELISA) kits (Enzyme-linked Biotechnology Co., Ltd., Shanghai, China) were employed to measure TNF-α and IL-6 concentrations in liver tissue and cells.

### 2.6 Histological assessment

The histology of liver tissue was assessed using HE staining. Liver samples were fixed in 4% formaldehyde, embedded in paraffin wax, sectioned at 5 μm, and stained with HE. Qualitative evaluation of hepatic lipid accumulation was conducted using ORO staining. Fresh 8-μm-thick frozen sections of liver tissue were prepared and stained with ORO following standard procedures. For cells, after washing, the HepG2 cells were fixed and then stained with ORO solution. Stained sections were examined and photographed using an Olympus BX53 microscope system. The ORO positive area was quantified utilizing ImageJ software (National Institutes of Health, Bethesda, MD, United States).

### 2.7 Transmission electron microscopy (TEM)

The mitochondrial ultrastructure of the liver was examined using TEM. The 1 mm^3^ tissue blocks were excised from fresh liver tissue and fixed in 1% OsO_4_ in 0.1 M phosphate buffer solution (pH 7.4) for 2 h at room temperature. Following fixation, the liver samples were dehydrated in an alcohol gradient and embedded in a mixture of acetone and 812 embedding agents in a 1:1 ratio for 2–4 h, followed by embedding in a 1:2 ratio overnight. The resin blocks were then sectioned to a thickness of 60–80 nm using an ultramicrotome (Leica UC7, Germany). Staining was carried out using a 2% uranium acetate saturated alcohol solution for 8 min and protected from light, followed by 2.6% lead citrate avoid CO_2_ staining for an additional 8 min. Finally, the cuprum grids were examined using a TEM (HT7800/HT7700, Japan) and images were captured.

### 2.8 Mitochondrial indicators involved in oxidative stress

Firstly, mitochondria were extracted from fresh liver tissue through the process of differential centrifugation and subsequently disrupted via ultrasonic crushing. Following this, the ATPase activity and ATP levels within the isolated mitochondria were quantified. Subsequently, the activities of SOD, GSH, GSH-Px, and the levels of MDA in both liver mitochondria and cell supernatants were assessed in accordance with the respective detection assay kit protocols (Jiancheng Bioengineering Institute, Nanjing, China). The protein concentration was determined using the BCA assay.

### 2.9 RNA extraction and real-time quantitative PCR (RT-qPCR) assay

Total RNA in liver tissue and HepG2 cells was extracted using the Trizol method following the standard protocol provided by Invitrogen (Carlsbad, CA, United States). Subsequently, the RNA was reverse transcribed into cDNA using the reverse transcription kit (Cat# CW 2020, Cowin Biotech Co., Ltd., Beijing, China) as per the manufacturer’s instructions. RT-qPCR was performed on the QuantStudio 5 Real-Time PCR System using the SYBR Green I PCR kit (Cat# CW2601, Cowin Biotech Co., Ltd., Beijing, China) following the supplier’s protocol. PCR amplification was conducted in a 20 μL reaction volume containing 10 μL of 2× UltraSYBR Mixture, 1 μL each of forward and reverse primers (10 μM), 1 μL of DNA template, and 7 μL of ddH_2_O. The amplification process commenced with an initial denaturation step at 95°C for 10 min, followed by 40 cycles of denaturation at 95°C for 10 s, annealing at 60°C for 30 s, extension at 72°C for 32 s, and a final step at 4°C. Relative expression of the target gene was calculated using the 2^−ΔΔCt^ method after normalization to GAPDH expression levels. The specific primer sequences utilized are provided in Table 1.

**TABLE 1:** Primers used in this study.

Primer name	Primer sequence (5′ to 3′)	Gene name
GAPDH-F GAPDH-R AMPK-F AMPK-R PGC-1α-F PGC-1α-R SIRT3-F SIRT3-R PPARγ-F PPARγ-R FGF21-F FGF21-R	TTGTCATGGGAGTGAACGAGA CAGGCAGTTGGTGGTACAGG TGCTTCATCTGTAGTCTCTGCTA CTGTGGTATCTGTGTTAGGTATGTT TATGGAGTGACATAGAGTGTGCT GTCGCTACACCACTTCAATCC TACAGAAATCAGTGCCCCGA GGTGGACACAAGAACTGCTG GGTTGACACAGAGATGCCAT GCTGGAGAAATCAACTGTGG GCCAAACACCGATTGGGGT GGCTCCAAATCTCCTTGGTAGTT	GAPDH AMPK PGC-1α SIRT3 PPARγ FGF21

### 2.10 Western blot

Proteins were extracted from frozen liver tissues or HepG2 cells using a lysis working solution containing a PMSF tablet in 10 mL of RIPA lysis buffer. Following grinding and centrifugation, the supernatant was collected for protein concentration determination using a BCA protein assay. Approximately 40 μg of protein samples were loaded into the wells of an SDS-PAGE gradient gel for electrophoresis at a constant voltage of 40 V for 30 min (concentration gel) or 100 V for 40 min (separation gel). The proteins were subsequently transferred to a polyvinyl difluoride membrane (Millipore, Boston, MA) for immunoblotting, followed by blocking with 5% non-fat milk for 2 h at room temperature. The membranes were then incubated overnight at 4°C with primary antibodies (Affinity Biosciences, Jiangsu, China) for anti-AMPK (1:1,000 dilution), anti-pAMPK (1:1,000), anti-SIRT3 (1:1,000), anti-PGC-1α, anti-PPARγ (1:1,000), and anti-FGF21 (1:1,000). The following day, the membranes underwent three washes with TBS-T and were subsequently incubated with horseradish peroxidase-conjugated secondary antibodies at a dilution of 1:5,000 for 1 h at room temperature. β-actin or Calnexin were utilized as internal reference proteins. Protein expression was detected utilizing the ChemiSignal™ Plus ECL, and protein bands were visualized with the ChemiScope 6000 Exp (Clinx Science Instruments Co. Ltd., Shanghai, China). The intensities of the target protein bands were quantified using Image J software (National Institutes of Health, Bethesda, MD, United States).

### 2.11 Statistical analysis

The experimental data were presented as mean ± standard deviation (SD) and statistical analyses were carried out using SPSS v26.0, with graph preparation conducted using GraphPad Prism 9.0.0. Prior to analysis, all data underwent tests for normal distribution and homogeneity of variance. For data meeting these criteria, differences among multiple groups were assessed using one-way analysis of variance followed by the least significant difference *post hoc* test. In cases where data did not meet assumptions of normality and homogeneity of variances, Kruskal-Wallis H non-parametric tests were utilized. Statistically significant differences were defined as *P* < 0.05.

## 3 Results

### 3.1 Analysis of CEL content in *Celastrus orbiculatus* Thunb. using HPLC

**FIGURE 1:**
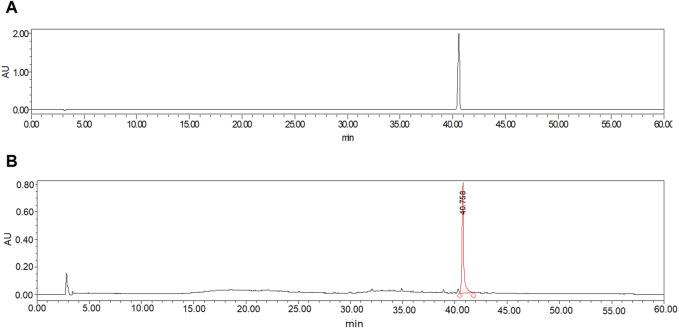
HPLC chromatogram of ethanolic extract of *Celastrus orbiculatus* Thunb. **(A)** CEL; **(B)** COT extract.

In this study, the ethanol heating reflux method was employed to obtain the COT extract. Then HPLC analysis of standard CEL and ethanolic extract of COT showed the presence of CEL (Figures 1A, B). The peak area of the COT extract was measured at 966,814,8, whereas the peak area of the standard sample was recorded at 221,126,36. Based on the standard curve constructed using a CEL standard solution, the concentration of CEL in the roots of COT was determined to be 0.9 mg/g.

### 3.2 COT extract and CEL attenuated hepatic steatosis and metabolic dysfunction in HFD-fed mice

**FIGURE 2:**
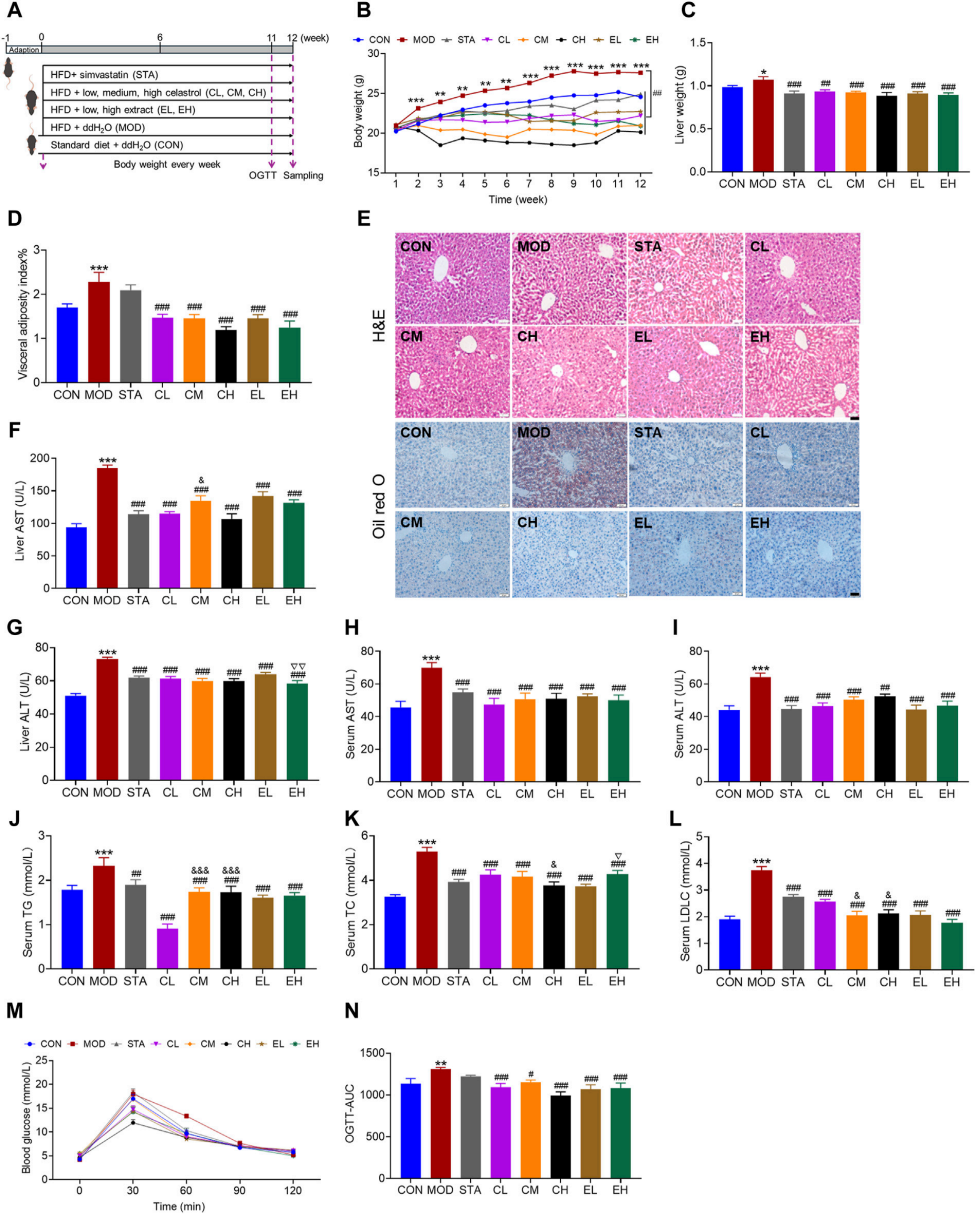
COT extract and CEL attenuated hepatic steatosis and metabolic dysfunction in HFD-fed mice. **(A)** Experiment design used to evaluate the role of COT extract and CEL against NAFLD; **(B)** Body weight recorded every week; **(C)** Liver weight and **(D)** visceral adiposity index at the end of the experiment; **(E)** Representative images of HE- and ORO-stained liver sections (×400 magnification, scale bar = 50 μm); **(F–I)** AST and ALT levels in livers and serum; **(J–L)** Levels of TG, TC and LDL-C in livers and/or serum (n = 8); **(M)** Blood glucose levels in OGTT (n = 6); **(N)** Summary of area under the curve (AUC) of the OGTT. Data are represented as mean ± SD. *P < 0.05, **P < 0.01, and ***P < 0.001 vs. the CON group; ^#^ *P* < 0.05, ^##^ *P* < 0.01, and ^###^ *P* < 0.001 vs. the MOD group; ^&^ *P* < 0.05 and ^&&&^ *P* < 0.001 vs. the CL group; ^▽^ *P* < 0.05 and ^▽▽^ *P* < 0.05 vs. the EL group.

In this study, NAFLD murine models were induced through HFD feeding (Figure 2A). As the duration of HFD feeding increased, mice exhibited a gradual increase in body weight. Compared to the CON group, mice fed with HFD showed a significant increase in body weight starting from the second week. Treatment with CEL and COT extract effectively attenuated the increase in body weight (Figure 2B). Additionally, liver weight and visceral adiposity index were significantly elevated in the HFD-fed groups but were reduced by CEL and COT extract treatment (Figures 2C, D).

The histological analysis of liver sections demonstrated that mice fed a HFD exhibited hepatic steatosis, disrupted lobular structure, and hepatic cord disorder, while mice treated with CEL or COT extract showed notable improvement in these parameters. ORO staining indicated extensive lipid accumulation in the MOD group, which was significantly reduced by CEL or COT extract treatment as evidenced by decreased positive red staining area (Figure 2E). Furthermore, the levels of ALT and AST in the liver and serum, as well as TG, TC, and LDL-C in the serum, were found to be significantly elevated in the MOD group compared to the CON group. However, supplementation with CEL or COT extract notably reversed these pathological changes as with simvastatin (Figures 2F–L). Meanwhile, for some parameters including liver AST, ALT, serum TC, TG and LDL-C, despite some variations among the groups treated with different dosages, a dose-dependent effect was not detected. Results from the OGTT demonstrated impaired glucose tolerance in NAFLD mice, with higher blood glucose levels observed after glucose administration, particularly at 60 min. Treatment with CEL or COT extract significantly improved these glucose tolerance levels, and the effect was better than simvastatin (Figures 2M, N). These findings suggest that CEL or COT extract has the potential to reverse liver injury and inhibit the progression of NAFLD.

### 3.3 COT extract and CEL improved the structure and function of liver mitochondria during NAFLD induced by HFD feeding in mice

**FIGURE 3:**
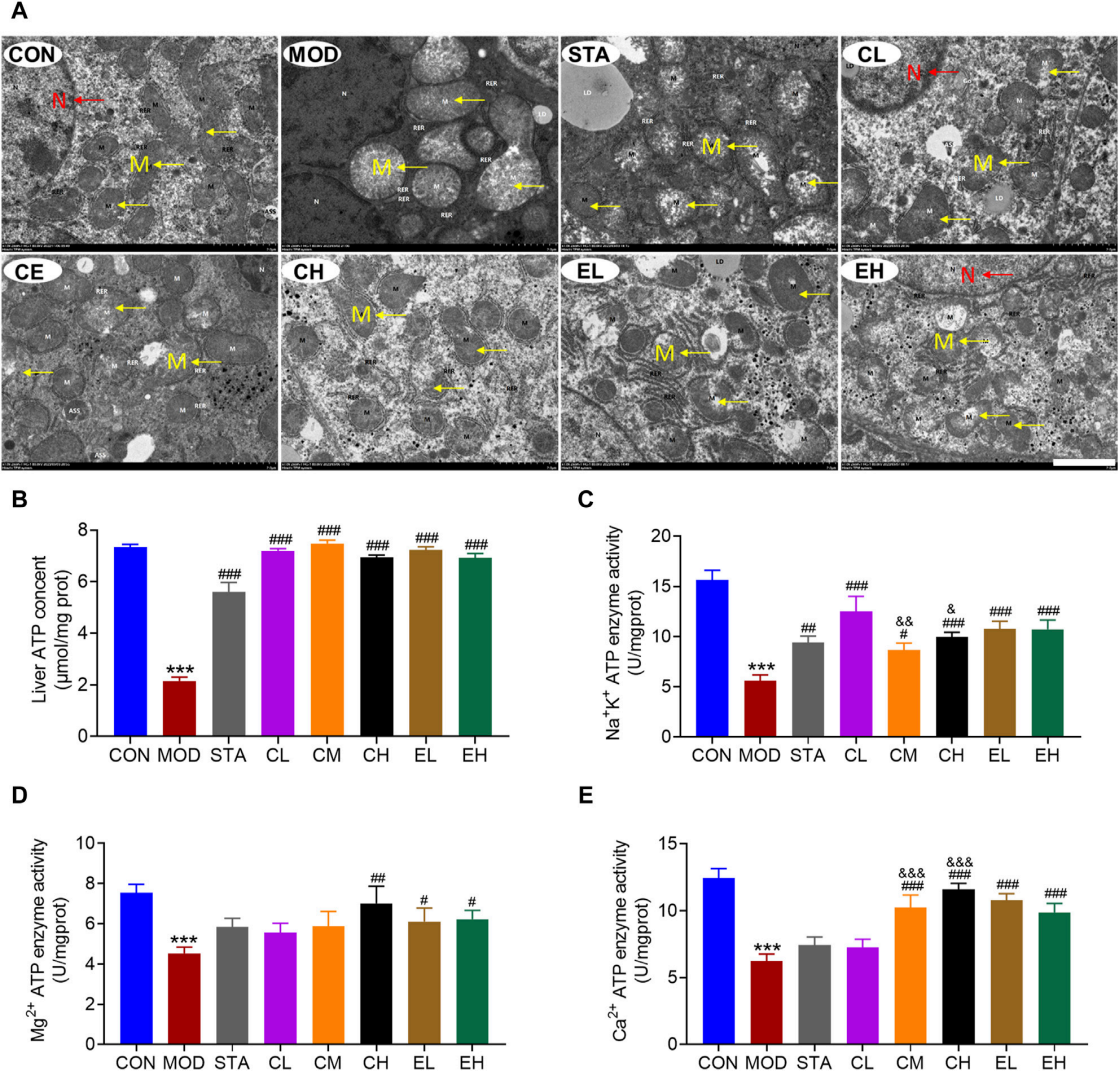
COT extract and CEL improved the structure and function of liver mitochondria during NAFLD induced by HFD feeding in mice. **(A)** Representative TEM images of mouse hepatocytes, M represents the mitochondria, while N represents the nucleus (×2 k magnification, scale bar = 2 μm); Activity of **(B)** Na^+^/K^+^ ATPases **(C)** Mg^2+^ ATPases and **(D)** Ca^2+^ ATPases in liver tissue (n = 8); **(E)** liver ATP content (n = 3). Data are represented as mean ± SD. ***P < 0.001 vs. the CON group; ^#^ *P* < 0.05, ^##^ *P* < 0.01, and ^###^ *P* < 0.001 vs. the MOD group; ^&^ *P* < 0.05, ^&&^ *P* < 0.05, and ^&&&^ *P* < 0.001 vs. the CL group.

The electron microscope analysis revealed that the liver tissue of mice in the CON group exhibited typical cellular morphology characterized by numerous organelles, minimal fatty degeneration, and absence of hepatic fibrosis. Mitochondria appeared as either ovoid or columnar structures with intact membranes and well-defined cristae. In contrast, liver samples from mice with NAFLD displayed pronounced inflammatory fibrosis and edema. Mitochondria in these samples exhibited severe swelling and enlargement, dissolution of the matrix within the membrane, reduction or absence of cristae, and vacuolation. Following treatment with simvastatin, CEL or COT extract, there was an improvement in cell and mitochondria morphology, characterized by non-significant fibrosis and hepatic steatosis, mild edema, some broken membrane, and noticeable cristae (Figure 3A).

In comparison to the CON group, the MOD group exhibited a significant decrease in ATP content, which was subsequently restored through CEL or COT extract administration (Figure 3B). The enzymatic activities of Na^+^-K^+^-ATP and Ca^2+^/Mg^2+^-ATP serve as indirect indicators of ATP production alterations. Our findings indicated a significant increase in the activities of these three enzymes following CEL or COT extract treatment (Figures 3C–E). For Na^+^-K^+^-ATP and Ca^2+^-ATP enzyme activity, high doses of CEL exerted better effects than low doses (Figures 3C, E). Given that ATP is primarily synthesized in mitochondria, reduced ATP levels may signify mitochondrial dysfunction in certain instances.

### 3.4 COT extract and CEL mitigated the oxidative stress and inflammation caused by HFD in mice

**FIGURE 4:**
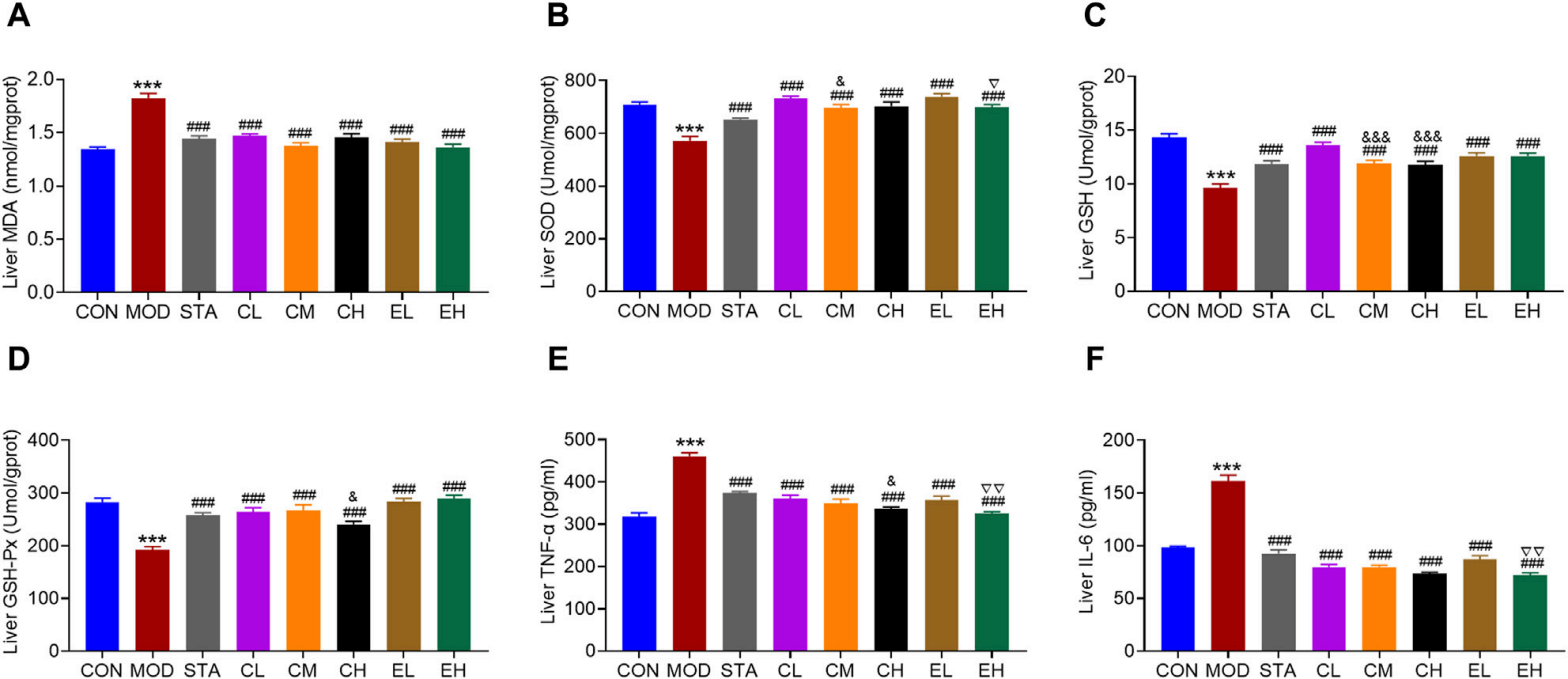
COT extract and CEL mitigated the oxidative stress and inflammation caused by HFD in mice. Changes in the levels of oxidative stress-related markers: **(A)** MDA **(B)** SOD **(C)** GSH, and **(D)** GSH-Px in liver tissue (n = 8); Levels of inflammatory factors **(E)** TNF-α and **(F)** IL-6 in liver tissue (n = 3). Data are represented as mean ± SD. ***P < 0.001 vs. the CON group; ^###^ *P* < 0.001 vs. the MOD group; ^&^ *P* < 0.05 and ^&&&^ *P* < 0.001 vs. the CL group; ^▽^ *P* < 0.05 and ^▽▽^ *P* < 0.05 vs. the EL group.

Numerous studies have indicated that an excess of oxidative stress and inflammation may contribute to the progression of NAFLD. In this study, levels of hepatic oxidative stress-related markers including MDA, SOD, GSH, and GSH-Px were assessed. The results depicted in Figures 4A–D demonstrated that a HFD led to elevated MDA levels and reduced SOD, GSH, and GSH-Px levels, which were significantly ameliorated by simvastatin and CEL/COT extract treatment, resulting in decreased MDA content and increased SOD, GSH, and GSH-Px levels. Furthermore, the impact of CEL or COT extract on production of the inflammatory cytokines was evaluated by measuring TNF-α and IL-6 levels. The findings indicated that HFD administration resulted in elevated levels of TNF-α and IL-6 in the liver, whereas treatment with CEL or COT extract demonstrated a notable inhibitory impact (Figures 4E, F). No dose linearity between low and high doses was revealed, and the effects were even slightly more distinct after low dose intake in comparison to the medium- and high-dose.

### 3.5 COT extract and CEL treatment inhibited HFD-induced increase in lipid levels possibly via regulating FGF21/AMPK/PGC-1α axis in the liver tissue of NAFLD mice

**FIGURE 5:**
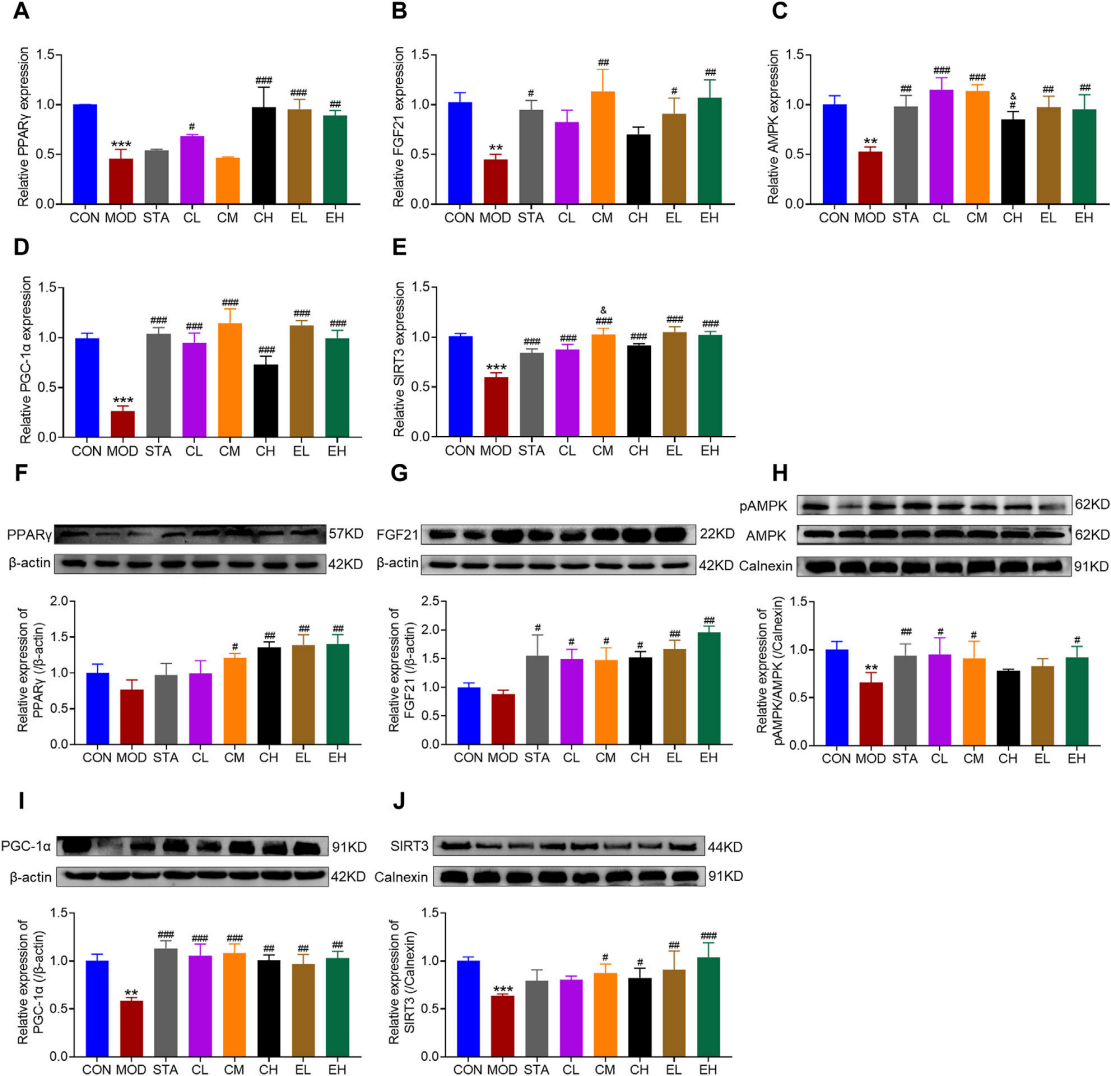
COT extract and CEL regulated FGF21/AMPK/PGC-1α signal pathway in HFD-fed mice. The mRNA expression of **(A)** PPARγ **(B)** FGF21 **(C)** p-AMPK **(D)** PGC-1α, and **(E)** SIRT3 in liver tissue by RT-qPCR analysis; The abundance of target protein involved in lipid metabolism and mitochondrial dysfunction: **(F)** PPARγ **(G)** FGF21 **(H)** p-AMPK **(I)** PGC-1α, and **(J)** SIRT3 in liver tissue. Experiments were repeated in triplicates. Data are represented as mean ± SD, n = 3. **p < 0.01 and ***p < 0.001 vs. the CON group; ^#^ *p* < 0.05, ^##^ *p* < 0.01, and ^###^ *p* < 0.001 vs. the MOD group; ^&^ *p* < 0.05 vs. the CL group.

Numerous studies have demonstrated a close association between mitochondrial dysfunction and the pathogenesis of NAFLD. Dysregulation of various proteins involved in mitochondrial function has been identified in NAFLD. The activation of FGF21/AMPK/PGC-1α signaling pathway in the liver plays a crucial role in suppressing hepatic steatosis and protecting mitochondrial function. Therefore, both RT-PCR and Western blot experiments were conducted to assess the impacts of CEL on hepatic FGF21, p-AMPK, PGC-1α, PPARγ and SIRT3 abundance in all mice. Compared to the CON group, HFD feeding led to decreased mRNA expression of FGF21, AMPK, PGC-1α, PPARγ, and SIRT3. The co-administration of CEL or COT extract significantly increased the expression of these genes, as illustrated in Figures 5A–E. Correspondingly, the protein levels of the aforementioned factors were also elevated following CEL or COT extract treatment, as shown in Figures 5F–J. Collectively, these findings suggested that CEL or COT extract activates the FGF21/AMPK/PGC-1α signaling pathway and enhances mitochondrial function in HFD-fed mice.

### 3.6 COT extract and CEL alleviated naol-induced intracellular lipid accumulation in HepG2 cells

**FIGURE 6:**
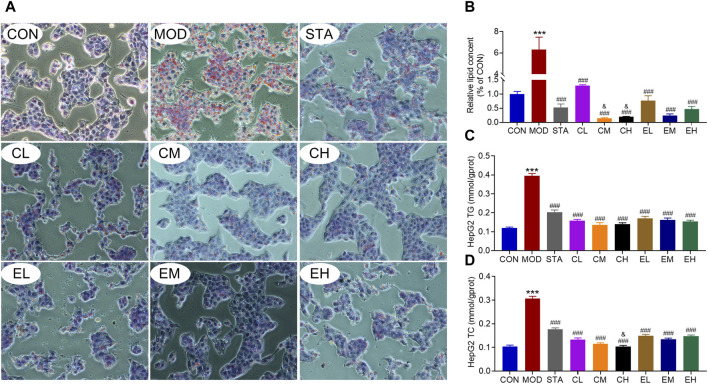
COT extract and CEL alleviated NaOL-induced intracellular lipid accumulation in HepG2 cells. **(A)** Representative images of ORO-stained HepG2 cells treated with NaOL with/without COT extract or CEL for 24 h (×400 magnification); **(B)** Quantitative analysis of ORO intensity; **(C, D)** Analysis of TG and TC content in HepG2 cells. Data are represented as mean ± SD, n = 3. ***P < 0.001 vs. the CON group; ^###^ *P* < 0.001 vs. the MOD group; ^&^ *P* < 0.05 vs. the CL group.

In order to validate the inhibitory impact of CEL on NAFLD, we utilized HepG2 cell lines to corroborate the findings *in vitro*. Lipid accumulation was assessed through Oil Red O staining. The results indicated a substantial presence of lipid droplets in NaOL-treated HepG2 cells, while CEL or COT extract treatment notably decreased hepatic lipid accumulation compared to the MOD group (Figures 6A, B). Additionally, levels of TG and TC in the cells were quantified. As depicted in Figures 6C, D, NaOL led to a significant increase in TG and TC levels, which were subsequently reduced by CEL or COT extract treatment. Meanwhile, high doses of COT extract exerted better effects than low doses. These findings suggest that COT extract or CEL may have the potential to inhibit lipid accumulation induced by NaOL.

### 3.7 COT extract and CEL attenuated NaOL-induced oxidative stress and inflammation *in vitro*

**FIGURE 7:**
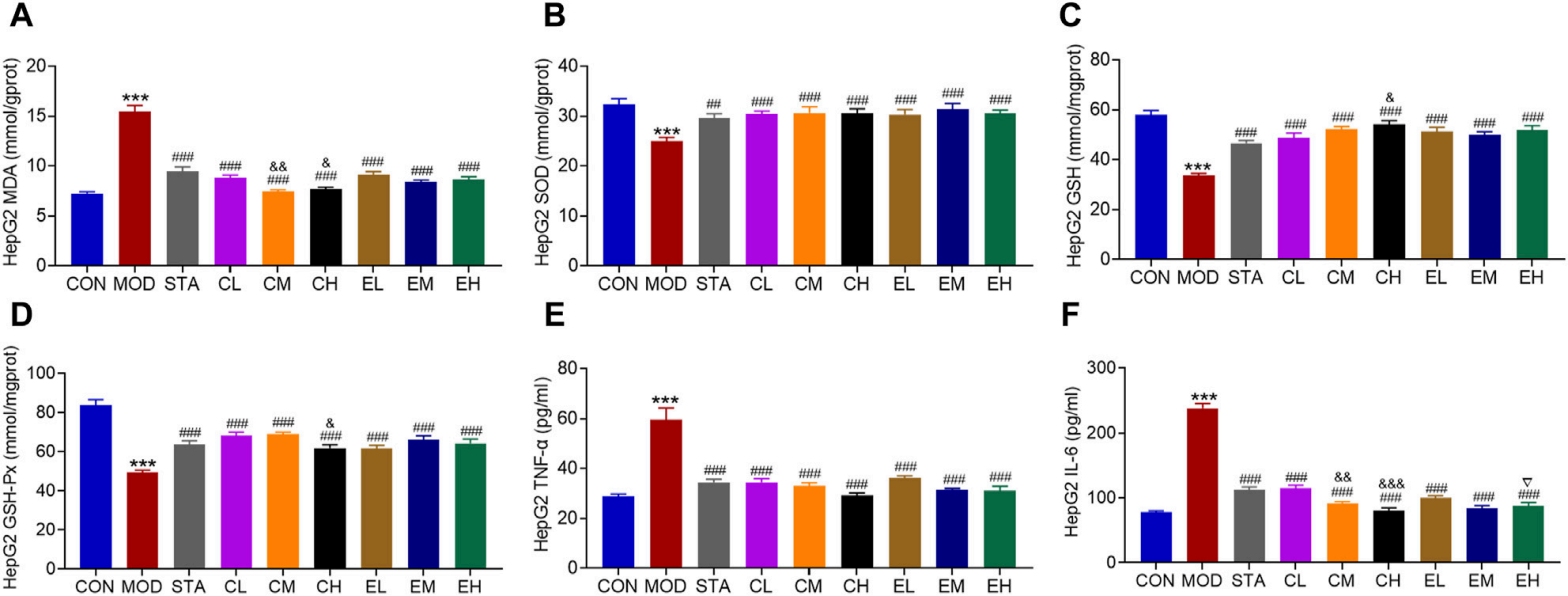
COT extract and CEL attenuated NaOL-induced oxidative stress and inflammation *in vitro*. Levels of **(A)** MDA **(B)** SOD **(C)** GSH **(D)** GSH-Px **(E)** TNFα, and **(F)** IL-6 in HepG2 cells. Data are represented as mean ± SD, n = 3. ***P < 0.001 vs. the CON group; ^###^ *P* < 0.001 vs. the MOD group; ^&^ *P* < 0.05 and ^&&^ *P* < 0.05 vs. the CL group; ^▽^ *P* < 0.05 vs. the EL group.

Similarly, the effects of CEL on intracellular oxidative stress-related indicators were evaluated. The co-culture of NaOL was found to increase the MDA content while significantly decreasing the levels of SOD, GSH, and GSH-Px in HepG2 cells. Treatment with CEL or COT extract notably improved antioxidant capacity by reducing MDA levels and increasing the concentrations of SOD, GSH, and GSH-Px, as depicted in Figures 7A–D. MDA, as a primary outcome of lipid peroxidation, has the potential to impair mitochondrial redox capacity and subsequently impact mitochondrial function. These findings suggested a potential association between the protective properties of CEL and mitochondrial function. Stimulation with NaOL led to an increase in TNF-α and IL-6 production, which was effectively attenuated by co-treatment with different concentrations of CEL or COT extract (Figures 7E, F). For some factors such as MDA, GSH and IL-6, there were significant differences between the doses, but with no apparent dose-related effect.

### 3.8 COT extract and CEL protected against mitochondrial dysfunction by regulating FGF21/AMPK/PGC-1Α signal pathway in HepG2 cells

**FIGURE 8:**
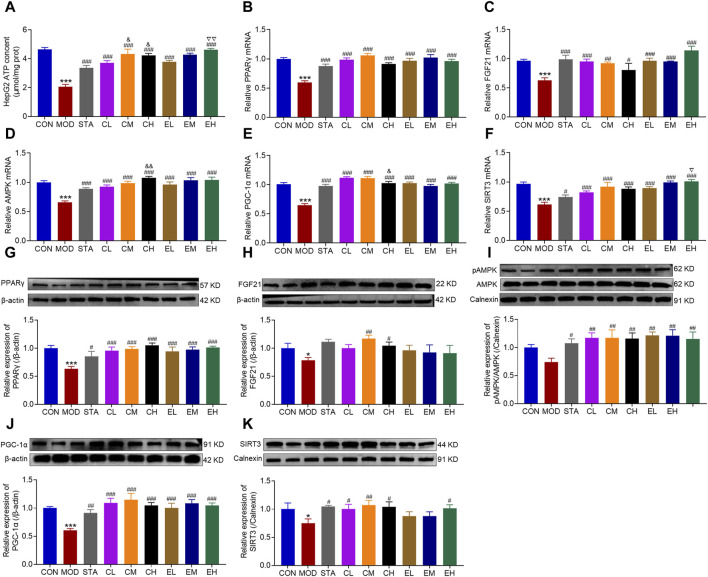
COT extract and CEL protected against mitochondrial dysfunction by regulating FGF21/AMPK/PGC-1α signal pathway in HepG2 cells. **(A)** ATP content in HepG2 cells. The mRNA expression of **(B)** PPARγ, **(C)** FGF21, **(D)** p-AMPK, **(E)** PGC-1α, and **(F)** SIRT3 in HepG2 cells; The abundance of target protein involved in lipid metabolism and mitochondrial dysfunction: **(G)** PPARγ, **(H)** FGF21, **(I)** p-AMPK, **(J)** PGC-1α, and **(K)** SIRT3. The experiments were repeated in triplicates. Data are represented as mean ± SD, n = 3. *P < 0.05 and ***P < 0.001 vs. the CON group; ^#^ *P* < 0.05, ^##^ *P* < 0.01, and ^###^ *P* < 0.001 vs. the MOD group; ^&^ *P* < 0.05 and ^&&^ *P* < 0.05 vs. the CL group; ^▽^ *P* < 0.05 and ^▽▽^ *P* < 0.01 vs. the EL group.

To confirm the findings of the experiment conducted *in vivo*, subsequent investigations at the RNA and protein levels were carried out *in vitro*. ATP content was reduced dramatically in the MOD group, while markedly increased with CEL or COT extract treatment (Figure 8A). The expression levels of molecules FGF21, AMPK, PGC-1α, PPARγ, and SIRT3 were significantly decreased in the HFD-fed group compared to the CON group (Figures 8B–F) and were remarkably restored by co-treatment with CEL or COT extract. Similarly, the protein levels of FGF21, AMPK, PGC-1α, PPARγ, and SIRT3 were reduced in the MOD group but were preserved with CEL or COT extract treatment (Figures 8G–K).

## 4 Discussion

NAFLD, ranked as the third leading cause of cancer-related mortality globally, has attracted considerable research and treatment focus due to its increasing prevalence. A comprehensive understanding of the underlying mechanisms associated with NAFLD is crucial for identifying novel therapeutic targets and strategies to effectively combat the disease. Numerous studies have highlighted the critical involvement of mitochondrial dysfunction, oxidative stress, and inflammation in the pathogenesis and progression of NAFLD to non-alcoholic steatohepatitis (NASH). The HFD-induced mouse model, exhibiting lipid metabolism disorders, insulin resistance, and oxidative stress, is considered an ideal NAFLD model due to its similarity to the pathogenesis observed in NAFLD patients (Huang et al., 2018). In this study, C57BL/6J mice were fed a HFD to induce hepatic steatosis, mimicking NAFLD, to investigate the therapeutic effects and mitochondrial-related mechanisms of COT against NAFLD in both animal and cell models. Results displayed that both COT extract and CEL effectively attenuated the progression of NAFLD by inhibiting hepatic lipid accumulation, oxidative stress, and inflammation. Moreover, FGF21/AMPK/PGC-1α signaling pathway was activated to alleviate mitochondrial damage. In spite of the complicated components of COT extract, our findings demonstrated that CEL is an essential component, as they both exhibited similar anti-NAFLD effects.

The liver plays a central role in maintaining lipid metabolism homeostasis by regulating lipid energy metabolism, conversion, and storage. Under normal conditions, lipid metabolism in the liver is balanced. However, prolonged consumption of high-fat and high-calorie diets can disrupt the equilibrium, leading to lipid overload and abnormal lipid metabolism. This can result in hepatic fat accumulation, excessive weight gain, and the development of NAFLD (Zhang et al., 2023). In this study, COT extract or CEL intervention in HFD-fed mice resulted in a reduction in body weight, potentially due to decreased liver weight and visceral adiposity index. Over the past dozen years, research has indicated that CEL may serve as a leptin sensitizer, leading to decreased body weight and showing promise as a potential pharmacological treatment for obesity (Liu et al., 2015). It is widely recognized that TG is the primary form of lipid stored in the fatty liver. Patients with lipid disorders often exhibit irregular lipid profiles, including elevated TC and/or TG (Tokgözoğlu and Libby, 2022). A prior study demonstrated a substantial decrease in body weight, subcutaneous and visceral fat mass, and hindered lipid droplet formation and accumulation in the liver of NAFLD mice after treatment with CEL. Moreover, biochemical analysis revealed a significant decrease in TG concentrations in both the liver and serum (Zhang et al., 2017). Previous studies have also reported the effects of CEL in inhibiting liver TG and TC deposition, as well as preventing hepatic steatosis to impede the progression of NAFLD (Han et al., 2018; Sun et al., 2020). In the current study, HFD feeding caused severe liver damage in NAFLD mice, characterized by numerous vacuoles in liver cells and elevated serum levels of TC, TG and LDL-C. CEL was found to decrease the degree of hepatic steatosis, as evidenced by a reduction in the number of fat vacuoles in the hepatic lobule, lower serum levels of lipid profile, and decreased positive ORO staining area (Figures 2, 6). Furthermore, the impaired glucose tolerance observed in NAFLD mice may contribute to the development of more severe hepatic steatosis, potentially due to elevated serum TG/TC levels and increased adipose tissue weight, which can impair glucose tolerance in mice (Diao et al., 2022). Administration of COT extract or CEL notably improved glucose tolerance in NAFLD mice (Figure 2).

Elevated levels of liver enzymes ALT and AST are widely recognized as diagnostic indicators of liver dysfunction, with ALT primarily localized in the cytoplasm and AST predominantly found in hepatocellular mitochondria. Consequently, serum AST levels will significantly increase in cases of severe hepatocyte damage (Diao et al., 2022). This study showed that both liver tissue and serum levels of AST and ALT were elevated in the MOD group compared to the CON group but were significantly reduced following treatment with COT extract or CEL (Figure 2). These results suggested that membrane structures and mitochondria were severely disrupted, resulting in the release of AST and ALT from hepatocytes into the bloodstream and subsequent elevation of serum ALT and AST concentrations. Overall, the study indicated that COT treatment may have potential benefits in preventing obesity, improving glycolipid metabolism, and mitigating hepatic steatosis.

It is well-established that excessive lipid accumulation can induce the production of oxygen-free radicals, resulting in lipid peroxidation, oxidative stress, and inflammatory reactions (Wang et al., 2022), all of which play significant roles in the development of NAFLD. Lipid peroxidation is initiated by ROS, leading to the formation of lipid peroxides (LPO) such as MDA and 4-hydroxynonena. These LPOs can be neutralized by antioxidants including GSH, glutathione peroxidase 4, SOD, and other molecules (Zhao et al., 2023). Hepatic lipid overload has been shown to trigger the generation of ROS, resulting in oxidative stress. Elevated levels of ROS can lead to oxidative alterations in cellular macromolecules (such as DNA, lipids, and proteins) and impair mitochondrial function, thereby promoting the initiation and progression of liver injury. Furthermore, the accumulation of fat and ROS signaling in the liver can enhance its susceptibility to inflammation (Chen et al., 2020). In the context of NAFLD animal models induced by HFD, researchers have highlighted the significant role of inflammatory cytokines such as TNF-α, interferon, and IL in the progression of NAFLD. Specifically, TNF-α has been found to inhibit the hepatic transport of cholesterol outside the liver while also promoting its accumulation within the organ. Additionally, TNF-α can stimulate fat breakdown, raise levels of free fatty acids, and subsequently increase the production of ROS in liver cells. This cascade of events exacerbates oxidative stress and contributes to damage in liver cells (Kodama et al., 2009). IL-6 is susceptible to production induced by TNF-α, which can worsen hepatic steatosis, insulin resistance, and inflammation in the pathogenesis of NAFLD, thereby facilitating the onset and progression of the disorder (Cengiz et al., 2014). Numerous studies have highlighted the efficacy of CEL as an antioxidant and anti-inflammatory substance in mitigating ROS, LPO, DNA oxidative damage, and inflammatory reactions (Allison et al., 2001; Shaker et al., 2014; Wang et al., 2020). Our findings demonstrated a significant reduction in MDA, TNF-α, and IL-6 levels, as well as an increase in SOD, GSH, and GHS-Px levels with supplementation of CEL or COT extract in both NAFLD mice and HepG2 cells (Figures 4, 7). The evidence strongly supported the notion that the administration of CEL or COT extract effectively improves abnormal lipid peroxidation by upregulating the expression of SOD, downregulating the production of ROS and LPO, and activating the nuclear factor erythroid-2 related factor/heme oxygenase-1 (Nrf-2/HO-1) pathway to mitigate oxidative stress and inflammation in NAFLD (Kim et al., 2013; Shaker et al., 2014). Additionally, studies have shown that CEL is effective in reducing inflammatory cell infiltration and hepatic fibrosis by lowering serum TC levels and hepatic lipid accumulation (Fan et al., 2022a).

The literature has extensively documented morphological and functional changes in mitochondria at different stages of NAFLD progression (Ajith, 2018). In NASH, approximately 40% of mitochondria exhibit structural abnormalities, leading to increased ROS production, resulting in lipid peroxidation of mitochondrial membranes, increased TNF-α expression, and impaired functionality (Echeverría et al., 2022). AMPK, a key metabolic energy sensor, plays a crucial role in maintaining energy balance by regulating mitochondrial biogenesis, fatty acid oxidation, and cell proliferation (Lin and Hardie, 2018). FGF21 and PGC-1α have been identified as downstream mediators of AMPK signaling. FGF21, a hepatokine primarily synthesized in the liver, exhibits dynamic contributions from the pancreas, adipose tissue, and skeletal muscle (Tillman and Rolph, 2020). It exerts regulatory effects on glucose metabolism in the liver and white adipose tissue, with elevated endogenous serum levels observed in patients with NAFLD, potentially offering protective benefits (Gaich et al., 2013; Barb et al., 2019). Studies have shown a decrease in hepatic FGF21 levels in obese insulin-resistant mice fed a HFD, a process characterized by a complex cascade involving the downregulation of AMPK and PGC-1α (Ortiz et al., 2020). PGC-1α functions as a key regulator of mitochondrial biogenesis and can be enhanced through AMPK phosphorylation (Li et al., 2020). Upon phosphorylation, PGC-1α translocates from the cytoplasm to the nucleus to initiate mitochondrial biogenesis (Dominy and Puigserver, 2013). Various animal investigations and clinical trials have demonstrated a reduction in AMPK activity in tissues such as the liver, muscles, and adipose tissue in chronic disease models or patients. Nevertheless, the administration of AMPK activators has been shown to substantially boost both AMPK and PGC-1α activity, leading to improvements in symptoms such as blood glucose levels, lipid profiles, and insulin resistance (Wang et al., 2010; Hardie, 2013). Activation of elements within the FGF21/AMPK/PGC-1α cascade is crucial in regulating mitochondrial energy metabolism and has attracted considerable attention as a potential therapeutic target for addressing metabolic disorders like NAFLD. Numerous plant-derived natural compounds have been utilized to modulate this signaling pathway and improve NAFLD by activating AMPK, including curcumin (Lee et al., 2019), Resveratrol (Baur et al., 2006), berberine (Sun et al., 2018), blueberry leaf polyphenols (Li et al., 2020) and punicalagin (Liu et al., 2021). Consistent with previous findings, our results revealed varying degrees of mitochondrial damage, characterized by swelling, reduced cristae, and vacuolization. Additionally, the decrease in ATP content and ATPase activity, along with the downregulation of FGF21, p-AMPK, and PGC-1α were observed in both HFD-induced liver injury and NaOL-induced hepatocyte steatosis. However, treatment with CEL or COT extract resulted in improved mitochondrial morphology, increased ATP content and ATPase activity, and upregulation of FGF21, p-AMPK, and PGC-1α levels compared to the untreated HFD group (Figures 3, 5, 8). It was demonstrated that PGC-1α is a downstream target of CEL-induced AMPK activation, indicating a potential regulatory role of CEL in the AMPK/PGC-1α signaling pathway. These findings suggested that the protective effects of CEL or COT extract against NAFLD may be mediated through their modulation of FGF21/AMPK/PGC-1α signaling pathway.

PPARγ, characterized by its predominant expression in adipose tissues, is known to play a crucial role in improving insulin resistance, inflammation, oxidative stress, and fibrosis, despite its potent adipogenesis-promoting effect (Chen et al., 2023). The exploration of natural PPARγ agonists has shown promising therapeutic efficacy in various liver diseases. Guo et al. (2013) demonstrated that puerarin effectively upregulates PPARγ expression and alleviates liver tissue fibrosis in a model of carbon tetrachloride-induced liver injury. Mahmoud et al. discovered that hesperidin can effectively upregulate PPARγ expression, thereby preventing liver injury (Pu et al., 2021). Our findings revealed a significant increase in PPARγ expression following CEL or COT extract administration compared to a NALFD model (Figures 5, 8), indicating potential beneficial effects that may shed new light on NAFLD.

Moreover, Sirtuin 3 (SIRT3) is localized within the mitochondrial compartment and plays a role in deacetylating mitochondrial proteins, thereby modulating energy metabolism and oxidative stress (Rardin et al., 2013). As a crucial downstream target of PGC-1α, SIRT3 collaborates with AMPK/PGC-1α signaling to reduce oxidative stress and cellular apoptosis (Zhang et al., 2016; Li et al., 2021). Previous investigations conducted in different disease conditions, such as spinal cord injury (Liu et al., 2017), myofascial pain syndrome (Ye et al., 2020), and type 2 diabetes skeletal muscle insulin resistance (Shi et al., 2015), have demonstrated that activation of the AMPK/PGC-1α/SIRT3 pathway can improve mitochondrial function and diminish mitochondrial dysfunction. SIRT3, a sirtuin highly expressed in the liver of mice, has been shown to enhance mitochondrial activity and ameliorate NAFLD by modulating ketogenesis, β-oxidation, mitogenesis, and antioxidant response systems (Nassir et al., 2018). Animal studies have demonstrated that mice fed with high-fat diet exhibit reduced SIRT3 activity, leading to impaired mitochondrial function, insulin resistance, and steatohepatitis (Choudhury et al., 2011; Hirschey et al., 2011). Our results suggested that SIRT3 expression was downregulated by a high-fat diet, but was restored by the co-administration of CEL or COT extract in both *in vitro* and *in vivo* studies, indicating a protective role of SIRT3 against fatty liver disease (Figures 5, 8). Mechanistically, the upregulation of SIRT3 expression reduced mitochondria oxidative stress and maintained mitochondrial function.

We have demonstrated the protective effects of COT extract or CEL on NAFLD and explored the mitochondrial protection mechanism through *in vivo* and *in vitro* experiments. Various drug concentrations were administered in this study, and although no statistically significant linear dose-response effects were detected, a dose-dependent relationship was evident for many parameters. One limitation of our study was the exclusive use of HepG2 cell lines instead of primary liver cells, which may not perfectly represent the metabolic characteristics of cells within the microenvironment of NAFLD individual. Furthermore, targeted gene knockdowns or knockouts in HepG2 cells and relevant animal models should be incorporated in future investigations. Additionally, the contribution of CEL to the action of the extract, as well as the potential role of other new monomer active components, must be further investigated due to the diverse chemical components present in drugs. The complex composition of the drug and its poor absorbability may pose challenges for clinical translation. Subsequent research efforts will focus on investigating the dosage, bioavailability, and efficacy of COT for clinical applications. Recent studies have emphasized the importance of lipid peroxidation and mitochondrial function in the newly identified cell death process termed ferroptosis. This study suggests that COT may influence lipid metabolism and mitochondrial function, indicating its potential role in regulating ferroptosis and warranting further research.

## 5 Conclusion

**FIGURE 9:**
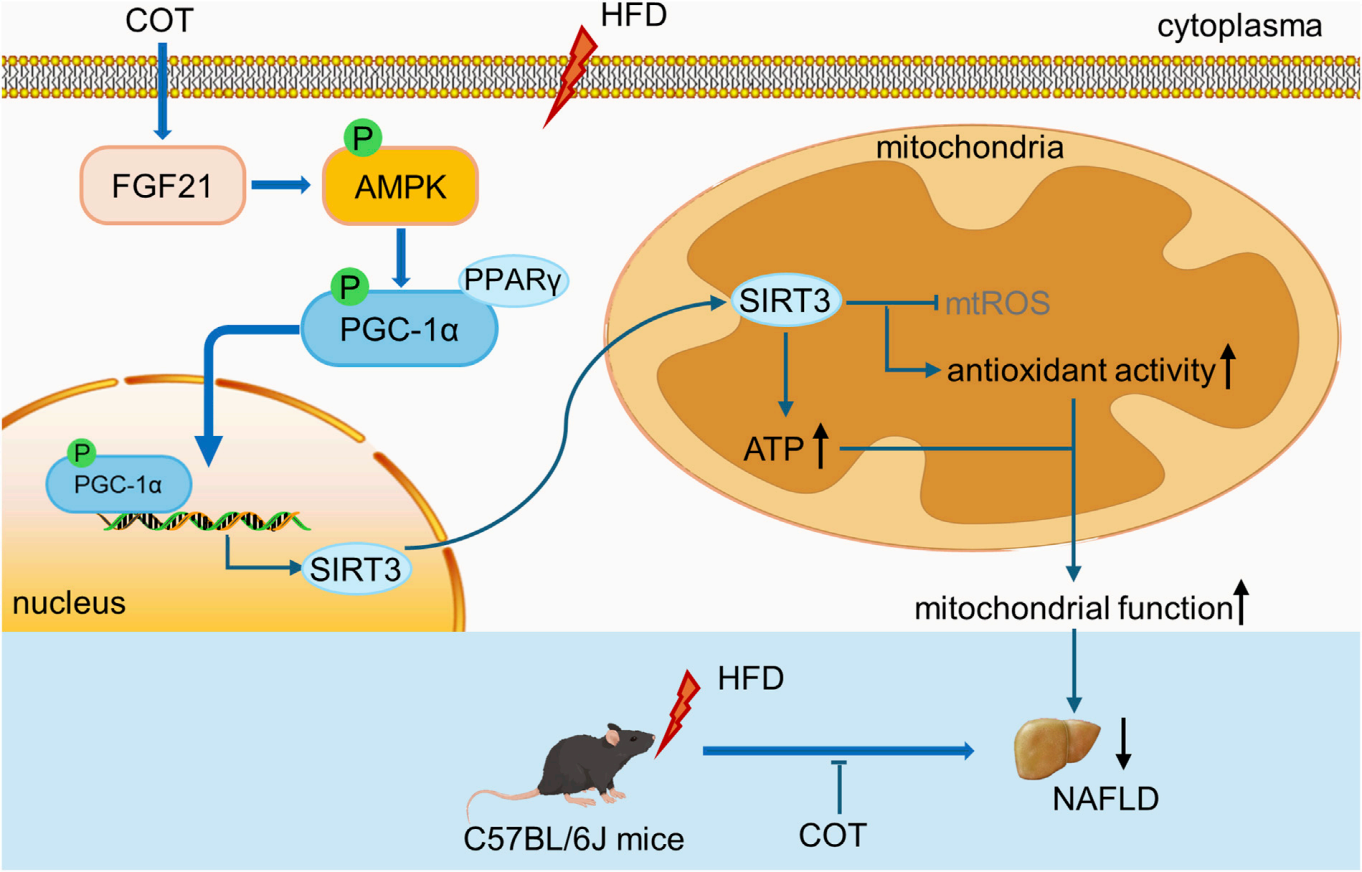
Schematic diagram showing the effects of COT extract and CEL on improving mitochondrial function in NAFLD via activating the FGF21/AMPK/PGC-1α signal pathway.

In summary, our study has successfully illustrated the protective properties of COT extract or CEL against NAFLD and elucidated the underlying mechanisms. The findings suggest that COT extract or CEL effectively ameliorated hepatic lipid accumulation, oxidative stress, and inflammation in both *in vivo* and *in vitro* models. These beneficial effects were linked to the upregulation of lipid metabolism and the activation of the FGF21/AMPK/PGC-1α pathway associated with mitochondrial generation (Figure 9). This research contributes valuable scientific evidence supporting the potential use of COT as a promising nutraceutical agent for managing NAFLD in the future.

## Data Availability

The original contributions presented in the study are included in the article/supplementary material, further inquiries can be directed to the corresponding author.
